# Salivary cortisone as potential predictor of occupational exposure to noise and related stress

**DOI:** 10.2478/aiht-2023-74-3785

**Published:** 2023-12-29

**Authors:** Roko Žaja, Sanja Stipičević, Milan Milošević, Andro Košec, Jakov Ajduk, Iva Kelava, Adrijana Zglavnik Baća, Marko Klarica, Mihael Ries

**Affiliations:** University of Zagreb School of Medicine, Zagreb, Croatia; Institute for Medical Research and Occupational Health, Zagreb, Croatia; University of Applied Health Sciences, Zagreb, Croatia

**Keywords:** cortisol, HPLC, psychosocial risks, hearing loss, HPLC, kortizol, nagluhost, psihosocijalni rizici

## Abstract

Salivary cortisone strongly correlates with serum cortisol, and since it is less invasive to measure salivary cortisone than serum cortisol and easier than to measure cortisol in saliva, as its concentrations are much lower, we wanted to compare salivary cortisone and cortisol levels as markers of noise-induced stress reaction. The study included 104 participants aged 19–30 years, 50 of whom were exposed to occupational noise ≥85 dB(A) and 54 non-exposed, control students. All participants took samples of their saliva with Salivette^®^ Cortisol synthetic swabs on three consecutive working days first thing in the morning. Salivary cortisone and cortisol levels were determined with high-performance liquid chromatography. In addition, they completed a 10-item Perceived Stress Scale (PSS-10) questionnaire, and occupationally noise-exposed participants also completed the Health and Safety Executive (HSE) questionnaire on occupational psychosocial risks. The exposed participants had significantly higher cortisone (P<0.001) and cortisol (P<0.001) levels than controls, and the correlation between cortisone and cortisol levels in the exposed participants was strong (ϱ =0.692, P<0.001), which suggests that salivary cortisone can replace cortisol measurements in saliva as a more reliable method than salivary cortisol and less invasive than serum cortisol. However, the level of perceived stress scored on PSS-10 in the exposed participants did not differ significantly from stress reported by controls, but correlated negatively with cortisone levels, which is contrary to our expectations and raises questions as to why.

## Abstract (sl)

Kortizon u slini snažno je povezan sa serumskim kortizolom, a kako je manje invazivno mjeriti kortizon u slini nego serumski kortizol te ga je lakše mjeriti nego kortizol u slini jer su mu koncentracije niske, htjeli smo usporediti koncentracije kortizona i kortizola kao biljega stresne reakcije izazvane bukom. U istraživanju su sudjelovala 104 ispitanika u dobi od 19 do 30 godina, od kojih je 50 izloženo buci ≥85 dB(A) na radnome mjestu, a 54 su neizloženi kontrolni studenti. Svi su sudionici prouzročili svoju slinu sintetičkim nosačima Salivette^®^ Cortisol tijekom triju uzastopnih radnih dana, odmah nakon jutarnjeg buđenja. Koncentracije kortizona i kortizola u slini bile su određene tekućinskom kromatografijom visoke djelotvornosti. Također, sudionici su ispunili Ljestvicu doživljenog stresa (PSS-10), a izloženi i upitnik *Health and Safety Executive* (HSE) o psihosocijalnim rizicima na radnome mjestu. Izloženi sudionici imali su značajno više koncentracije kortizona (P<0,001) i kortizola (P<0,001) nego kontrolni, te je korelacija između koncentracija kortizona i kortizola u izloženih sudionika bila jaka (ϱ=0,692, P<0,001), što upućuje na to da određivanje kortizona u slini može zamijeniti određivanje kortizola u slini, i to kao pouzdanija metoda od određivanja kortizola u slini te kao manje invazivna od određivanja serumskoga kortizola. Razina doživljenog stresa određena ljestvicom u izloženih sudionika nije se značajno razlikovala od razine stresa u kontrolnih sudionika, ali je bila negativno povezana s koncentracijama kortizona, što je suprotno našim očekivanjima te otvara nova pitanja.

The latest World Report on Hearing published by the World Health Organization (1) provides a thorough discussion about the development of noise-induced hearing loss (NIHL), including the hidden hearing loss (HHL), which precedes it and is undetectable by pure tone audiometry. Both have become a common disorder among young adults due to ever increasing occupational and recreational exposure to noise (1, 2, 3, 4). HHL entails the destruction of synapses between the cochlear nerve and hair cells and is manifested by tinnitus, hyperacusis, and difficulties in understanding speech in noise (5, 6).

Common non-auditory effects of exposure to noise include annoyance, although the certainty of evidence is low (7, 8). Noise annoyance has been reported to affect the hypothalamus-pituitary-adrenal (HPA) axis (9) and consequently higher cortisol levels in the serum and saliva (10, 11). Cortisol is even higher when noise and stressful thoughts/feelings are combined, as they seem to act synergistically (12, 13, 14). In salivary glands, free serum cortisol is rapidly converted into cortisone, which is more concentrated than salivary cortisol and correlates more strongly with serum cortisol than salivary cortisol (15, 16, 17). In spite of that, salivary cortisol is still one of the most studied metabolic markers of noise-induced stress reactions (8) as it accurately reflects serum cortisol levels (18, 19). Optimally, it is measured with liquid chromatography with tandem mass spectrometry (LC-MS/MS) (17, 20, 21), but De Palo et al. (22) have developed and validated a high-performance liquid chromatography (HPLC) method that can measure both salivary cortisone and cortisol levels at the same time.

The aim of our cross-sectional study was to use this method to establish the relationship between salivary cortisone and cortisol levels and their relationship with noise-induced stress.

## PARTICIPANTS AND METHODS

Participants consisting of occupationally exposed workers and university students as controls were recruited between May 2021 and March 2023. Those who did not report current use of oral or intranasal corticosteroids or previous sudden hearing loss, chronic otitis media, ear surgery, or vertigo associated with hearing loss and tinnitus, underwent audiological examination and risk assessment for noise-induced hearing loss at the Sestre milosrdnice University Hospital Department of Audiology and Phoniatrics, Zagreb, Croatia. Audiological examination included otoscopy, tympanometry, and pure-tone audiometry. Participants who had normal unilateral or bilateral findings (tympanogram with a peak pressure in the middle ear of ±50 daPa at 226 Hz and a middle ear compliance of 0.3 to 1.3 mL; audiogram with a hearing threshold >20 dB in a range from 0.25 to 8 kHz and threshold difference <15 dB between adjacent 3 to 6 kHz frequencies, and no more than one air-bone gap (<15 dB on a single frequency) proceeded to the next step, while one worker and two students did not.

The remaining candidates then completed the Megerson's questionnaire on previous noise exposure (23). The questionnaire consists of three multiple-choice questions regarding noise exposure over the previous year. The first question establishes the frequency of exposure to firearms impulse noise while the remaining two establish the frequency of exposure to intense noises that required shouting or speaking in a raised tone to be heard at arm's length at the workplace (second question) or outside the workplace (third question). The answers to the first question were scored as follows: *never* – 0 points; *every few months* – 3 points; *monthly* – 6 points; *weekly* – 9 points; and *daily* – 12 points. The answers to the second and third question were scored as follows: *never* – 0 points; *every few months* – 1 point; *monthly* – 2 points; *weekly* – 3 points; and *daily* – 4 points. The total level of risk of noise-induced hearing loss was calculated as the sum of all points.

Students with high (5, 6, 7, 8, 9, 10, 11, 12, 13, 14, 15, 16, 17, 18, 19, 20) risk of noise-induced hearing loss were excluded from further study (N=2) to remove bias. There were no workers with low (0–4) risk who met this exclusion criterion.

Eventually, the study included 104 participants (60 men and 44 women) aged 19–30 years, 50 of whom were manufacture and construction workers (N=21) or professional musicians (N=29) exposed to an average occupational noise of ≥85 dB(A) for at least one year before joining the study. According to their own claim and/or accounts by their occupational safety and health officers they were not occupationally exposed to ototoxic chemicals (e.g. carbon disulphide, xylene, or styrene). The unexposed, control group consisted of 54 university students.

Compared to controls (24.0 years, IQR 24.0–25.0 years), the worker group (27.0, IQR 23.8–29.0 years) was significantly older (U=773.5, z=−3.8, P<0.001) and had significantly more men (34 of 50 vs 26 of 54; P=0.041).

All participants signed informed consent, and the study was approved by the Ethics Committee of the Zagreb University School of Medicine.

### Questionnaires

All participants completed the Croatian version of the 10-item Perceived Stress Scale (PSS-10) questionnaire assessing psychological stress over the month preceding their participation in the study (24). The questionnaire consists of 10 multiple-choice questions about the frequency of stress-related thoughts and feelings scored on a Likert scale from 0 to 4. Items four, five, seven, and eight are scored inversely. The total level of self-perceived stress is the sum of all points.

Workers, in addition, completed the Croatian version of the Health and Safety Executive (HSE) questionnaire assessing occupational psychosocial risks (25). The questionnaire consists of 35 items with multiple-choice questions about the frequency of unawareness of organisational changes, role ambiguity, problematic relationships, lack of co-worker and managerial support, lack of control, and high demands. The answers are scored on a Likert scale from 1 to 5. The probability of exposure to a particular occupational psychosocial risk equals the mean of points in the above domains. It can be low (1.0 to 2.9), moderate (3.0 to 3.9), or high (4.0 to 5.0).

Both measures of psychological stress were used to see if they would correlate with salivary cortisone/cortisol and thus confound our findings about correlations between exposure to noise and salivary cortisol/cortisone.

### Saliva collection and cortisol and cortisone measurements

Each participant sampled saliva at home using Salivette^®^ Cortisol swabs (Sarstedt, Nümbrecht, Germany) once a day for three days in a row, right after waking up in the morning and before taking breakfast or brushing the teeth. They were asked to hold the swab in their mouth for three minutes, return it to its tube, and store in a refrigerator at 4–8 °C. After the third sampling, the participants delivered three tubes to the Institute for Medical Research and Occupational Health, Zagreb, Croatia. The tubes were immediately centrifuged at 425 *g* for 10 min and stored at −18 °C until analysis (for 3–73 days).

### Chemicals and standard solutions

Analytical 98 % purity cortisol (CAS No. 50-23-7) and cortisone (CAS No. 53-06-5), and internal standard 6α-methylprednisolone (CAS No. 83-43-2) were the products of Sigma-Aldrich^®^ (St. Louis, MO, USA). Solvents LiChrosolv^®^ methanol and LiChrosolv^®^ acetonitrile were supplied by J. T. Baker^®^ (Deventer, the Netherlands), while ChromaSolv™ Plus acetone and SupraSolv^®^ diethyl ether were supplied by Merck^®^ (Darmstadt, Germany). All solvents were of HPLC or gas chromatography (GC) analytical grade. Ultrapure water was obtained with a Millipore^®^ purification system (Bedford, MA, USA).

Individual stock solutions (1 mg/mL) were prepared in methanol and stored at −18 °C. Calibration standards, containing a mixture of cortisol and cortisone with concentrations of both molecules in the 50–500 nmol/L range, and the internal standard with constant concentration at 500 nmol/L were prepared from stock solutions diluted in methanol:water (1:1, v/v) right before analysis. A spike solution containing cortisol and cortisone for method efficiency test as well as the internal standard solution for spiking the real and fortified saliva samples were prepared from stock solutions further diluted in water. The internal standard solution in water was kept at −18 °C for no longer than two months.

### Sample preparation

Once the tubes with saliva thawed to room temperature, they were centrifuged again. The extraction followed the procedure described by De Palo et al. (22). From each of the three tubes collected per participant we combined 1.0 mL aliquots of supernatants, divided them in two equal subsamples (totalling 1.5 mL), and spiked them with 100 μL of the internal standard solution to the final concentration of 50 nmol/L. Each subsample was subjected to solid-phase extraction (SPE) using a 100 mg Discovery^®^ DSC-18 cartridge (Supelco^®^, Bellefonte, PA, USA) previously conditioned with 1.5 mL of methanol and then 1.5 mL of water. Impurities retained on the SPE sorbent were washed with 0.5 mL of water and 0.5 mL of acetone:water (1:4, v/v) before elution with 2.0 mL of diethyl ether. The extract was filtered through a 0.2 μm polytetrafluoroethylene filter, dried under a gentle stream of nitrogen, and reconstituted with 150 μL of methanol:water (1:1, *v*/*v*, enrichment factor of 10).

Analytical performance was tested by analysing steroid-spiked saliva samples, which were obtained by purifying control saliva from native steroids using the 250 mg Supelclean™ ENVI-Carb cartridge (Supelco^®^) and then by adding known amounts of cortisol and cortisone to the final concentrations of 10, 25, or 50 nmol/L of each analyte. Further analysis of the spiked samples followed the procedure described for real samples.

### HPLC analysis

Salivary cortisol and cortisone levels were measured on a Varian^®^ HPLC-UV/DAD (Walnut Creek, CA, USA). The system consisted of ProStar 230 solvent delivery unit, ProStar 410 autosampler, and ProStar 330 photodiode array detector. High selectivity was ensured with a Supelco Discovery^®^ HS F5 (15 cm × 2.1 mm, 5 μm) chromatographic column and gradient elution with acetonitrile and water according to the following programme: 20–40 % acetonitrile in 6 min followed by isocratic elution for 2 min, then gradient elution with 40–20 % acetonitrile in 2 min, and final isocratic elution for 10 min. The total run time at a flow rate of 0.25 mL/min was 20 min, including column conditioning before the next run. The injection volume was 50 μL, and detection was set to 245 nm.

Linearity was determined with correlation coefficients, which were higher than 0.999 for both steroids in the range from 50 to 500 nmol/L. The average recovery for cortisol and cortisone at levels ranging from 10 to 50 nmol/L exceeded 80 and 87 %, respectively, with an intra-assay imprecision (relative standard deviation, N=5) below 10 % and a limit of quantification of 5.5 nmol/L for both analytes.

### Statistical analysis

We used descriptive and inferential statistics for data analysis. Continuous data are shown as medians, while categorical data as counts and percentages. Depending on data distribution we used non-parametric chi-squared test for categorical variables, Mann-Whitney *U* test for continuous variables, and Spearman's rank correlation for salivary cortisone and cortisol levels, reports of stress levels, and occupational psychosocial risks. P values below 0.05 are considered significant. All statistics were run on IBM SPSS Statistics for Windows, version 25.0 (IBM Corp., Armonk, NY, USA).

## RESULTS

Table 1 shows that participants occupationally exposed to noise had significantly higher cortisone (U=792.0, z=−3.6, P<0.001) and cortisol (U=270.5, z=−4.4, P<0.001) levels than controls. Furthermore, cortisone and cortisol levels correlate significantly in the exposed participants (ϱ=0.692, P<0.001) and marginally in controls (ϱ=0.317, P=0.052). Cortisone levels were determined in all participants, while cortisol was determined in 35 (of 50) exposed and 38 (of 54) control participants.

**Table 1:** Salivary cortisone and cortisol levels among noise-exposed (N=50) and control participants (N=54)

	**Cortisone**		**Cortisol**	
	**Exposed**	**Control**	**Exposed**	**Control**
Median (nmol/L)	32.4^*^	21.2	8.7^*^	6.8
Interquartile range (nmol/L)	18.5–49.8	18.0–23.6	6.9–11.2	6.1–7.1

* significantly higher than control (P<0.001; Mann-Whitney *U* test)

However, the two groups do not differ significantly in perceived stress scores (exposed: 17.0, IQR 12.0–22.0 vs controls: 15.0, IQR 11.0–20.25; U=1205.5, z=−0.9; P=0.346), and the exposed participants show a significant negative correlation between perceived stress and cortisone (Table 2).

**Table 2:** Correlations between perceived stress (PSS-10 questionnaire) and cortisone and cortisol levels among noise-exposed (N=50) and control (N=54) participants

	**Cortisone**		**Cortisol**	
	**Exposed**	**Control**	**Exposed**	**Control**
Spearman's rho	−0.394	0.142	−0.220	0.176
P	**0.019** ^*^	0.395	0.125	0.204

* significant correlation at P<0.05

As for the occupational psychosocial risk assessment in participants occupationally exposed to noise, we found no correlations between the seven measured domains and cortisone or cortisol levels (Table 3).

**Table 3:** Correlations between the seven HSE domains and cortisone or cortisol levels in occupationally noise-exposed participants (N=50)

**HSE domains**	**Risk probability (median; IQR)**	**Cortisone**		**Cortisol**	
		**Spearman's rho**	**P**	**Spearman's rho**	**P**
Unawareness of changes	Moderate (3.7; 3.3–4.0)	0.109	0.451	−0.013	0.939
Role ambiguity	High (4.8; 4.4–5.0)	−0.188	0.190	−0.098	0.574
Problematic relationships	Low (1.8; 1.5–2.5)	0.053	0.714	0.074	0.714
Lack of co-worker support	High (4.0; 3.5–4.8)	0.124	0.391	0.001	0.997
Lack of managerial support	High (4.2; 3.4–4.5)	0.078	0.590	−0.088	0.617
Lack of control	Moderate (3.8; 3.5–4.2)	−0.077	0.597	−0.086	0.623
High demands	Low (2.4; 2.1–2.9)	−0.039	0.789	0.118	0.501

## DISCUSSION

Our study confirms that salivary cortisone measurements with HPLC can replace cortisol, as they are more reliable, considering that the method is less sensitive to low cortisol concentrations in saliva (17, 20) and the two measurements correlate (P<0.001 in noise-exposed participants and P=0.052 in controls). This correlation arises from the same metabolic pathway of cortisol and cortisone in response to noise (15) and higher salivary levels in the exposed participants.

Furthermore, our findings confirm the association between morning salivary cortisone and noise exposure, which is in line with reports of higher salivary cortisol with aircraft noise exposure in women (26) or men (27).

Much to our surprise, one of the key findings of our study is the significant *negative* correlation between the PSS-10 stress perception and salivary cortisone in the noise-exposed participants, which has not been reported earlier. Both the PSS-10 and the HSE questionnaires were run to exclude psychological stress as the confounding factor, as we expected to find no correlation between psychological stress and salivary cortisone/cortisol. No correlation would confirm that the main stressor is noise. As we did not observe any determinants that could explain the negative correlation, we assume that it may be owed to insufficient amount of sleep the night before sampling (28). However, the level of perceived stress in the exposed participants was within the expected values for age (24), did not significantly differ from controls, and their scores in HSE domains of occupational psychosocial risks did not correlate with higher cortisone (or cortisol) levels. Considering the low cortisol levels in the saliva and some reports of no correlation between perceived stress and salivary cortisol (29, 30) as opposed to serum or urinary cortisol (31, 32), this makes sense, but does not explain why the same is true for salivary cortisone.

## CONCLUSION

We believe that some of the questions raised by our study stem from its limitations. The first is that noise exposure was estimated based on work documentation and self-assessments instead of on-site measurements. The second is that the exposed participants experienced different types of occupational noise, seeing that half the group are musicians. The third is that participants took their own saliva samples at home and that HPLC may not be the best method for salivary cortisone and cortisol determination. Finally, there are inherent limitations to the psychological stress questionnaires used, as they require one to recall past experiences, and the HSE questionnaire was more difficult to apply to musicians than the rest of the exposed worker group. For one, professional musicians are more autonomous in their job and have a more flexible schedule.

## CONCLUSION

Even with these limitations, our study clearly shows that salivary cortisone can replace cortisol, as evidenced by the strong correlation between cortisone and cortisol levels. Further investigations should include a larger number of exposed participants, consider chromatographic methods with greater sensitivity and specificity, and examine a correlation between salivary cortisone and HHL audiological signs to improve NIHL prediction.
